# Supplementary material for: Trichoplein binds PCM1 and controls endothelial cell function by regulating autophagy
Source: EMBO Rep. 2020 Apr 26;21(7):e48192. doi: 10.15252/embr.201948192 (PMC7332983; doi:10.15252/embr.201948192)
Supplement: Supplementary file 1 — Appendix [file EMBR-21-e48192-s001.pdf]

Appendix for "Trichoplein binds PCM1 and controls endothelial cell function by regulating autophagy**"**

Table of Content

Appendix Figure S1……………………………………………………………page 2

Appendix Figure S2……………………………………………………………page 4

Appendix Figure S3…………………………………………………………....page 5

Appendix Figure S4……………………………………………………………page 6

A

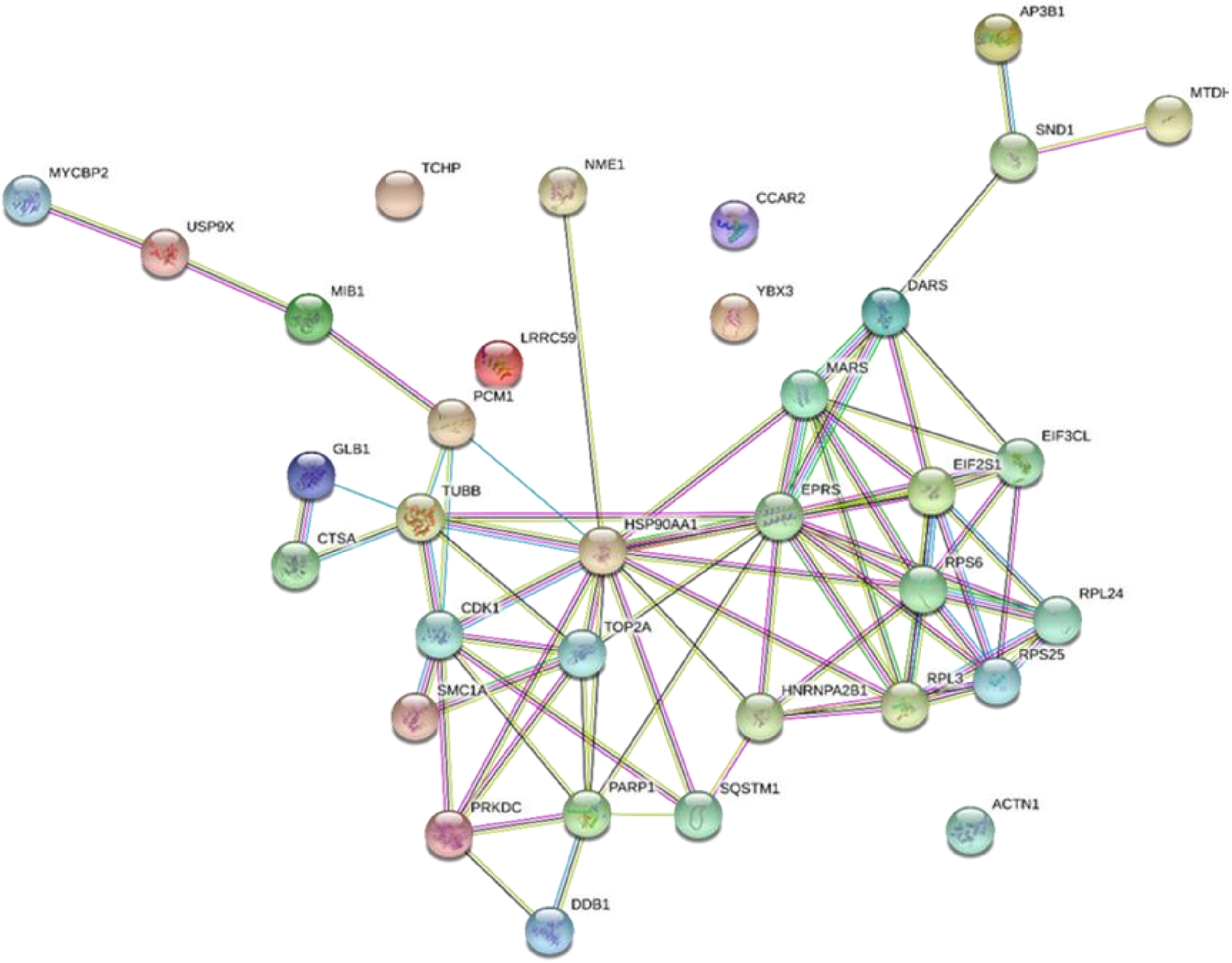

AP3B1
MTDH
SND1
MYCBP2
TCHP
NME1
CCAR2
USP9X
MIB1
YBX3
DARS
LRRC59
MARS
PCM1
EIF3CL
GLB1
EIF2S1
TUBB
EPRS
HSP90AA1
CTSA
RPS6
RPL24
CDK1
TOP2A
RPS25
SMC1A
HNRNPA2B1
RPL3
PARP1
SQSTM1
PRKDC
ACTN1
DDB1

B

Protein models:

TCHP Δ1

Model #01	File	Built with	Oligo-State	Ligands	GMQE	QMEAN
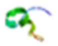	PDB	ProMod3 Version 1.3.0.	monomer	None	0.02	-2.31

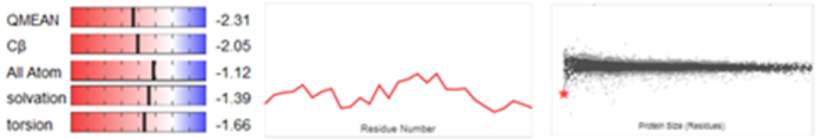

Template	Seq Identity	Oligo-state	Found by	Method	Resolution	Seq Similarity	Range	Coverage	Description
1xox.1.A	32.14	monomer	HHblits	NMR	NA	0.36	1 - 29	0.06	Apoptosis inhibitor survivin

Ligand	Added to Model	Description
ZN	✕ - Binding site not conserved.	ZINC ION
ZN	✕ - Binding site not conserved.	ZINC ION

TCHP Δ1,2

Model #01	File	Built with	Oligo-State	Ligands	GMQE	QMEAN
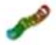	PDB	ProMod3 Version 1.3.0.	monomer	None	0.09	2.26

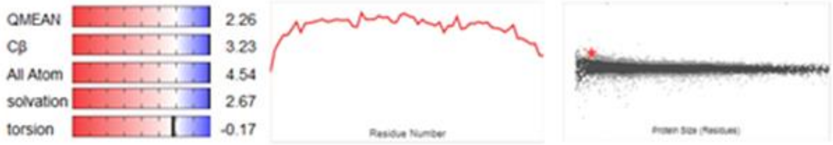

Template	Seq Identity	Oligo-state	Found by	Method	Resolution	Seq Similarity	Range	Coverage	Description
6fkh.1.X	14.06	monomer	HHblits	EM	NA	0.28	240 - 303	0.18	ATP synthase subunit b', chloroplastic

Appendix Figure S1: Protein network and TCHP mutants analysis

A, TCHP protein interaction network analysis using STRING software. **B**, The SWISS-MODEL (https://swissmodel.expasy.org/) template library (SMTL version 18-07-2019, PDB realese 12-7-2019) was searched with BLAST (Camacho, C et al. 2009) and HHBlits (Remmert et al. 2012) for evolutionary related structures matching the target sequence for TCHP Δ1 and TCHP Δ1,2. Top-hit homologues protein were Survivin for TCHP Δ1 and ATP synthase subunit b for TCHP Δ1,2

A B

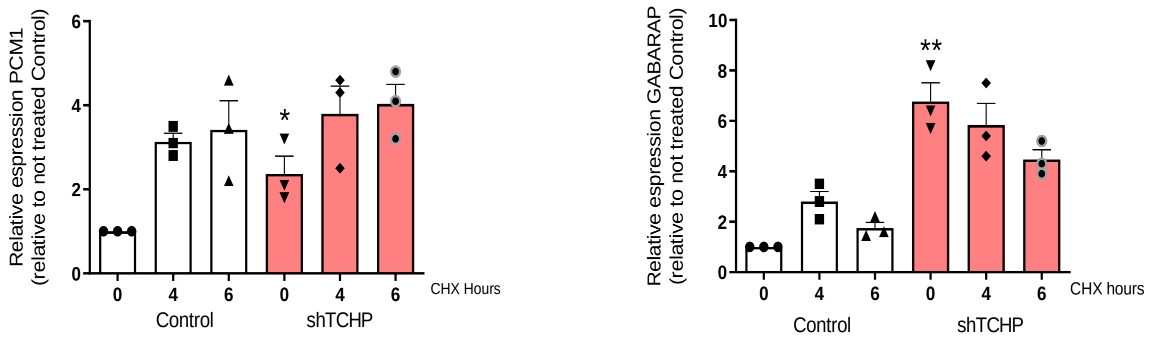

Appendix Figure S2: PCM1 and GABARAP expression

Expression of **A**, PCM1 and **B**, GABARAP in control or TCHP knock-down HUVECs were subjected to cycloheximide (CHX) for the indicated number of hours (A: one-way ANOVA, *p = 0.0325 vs control 0 hour; B: one-way ANOVA, **p = 0.0015 vs control 0 hour).

Data information: statistical analyses were performed on at least three independent experiments. Data are represented as mean ± SD.

A

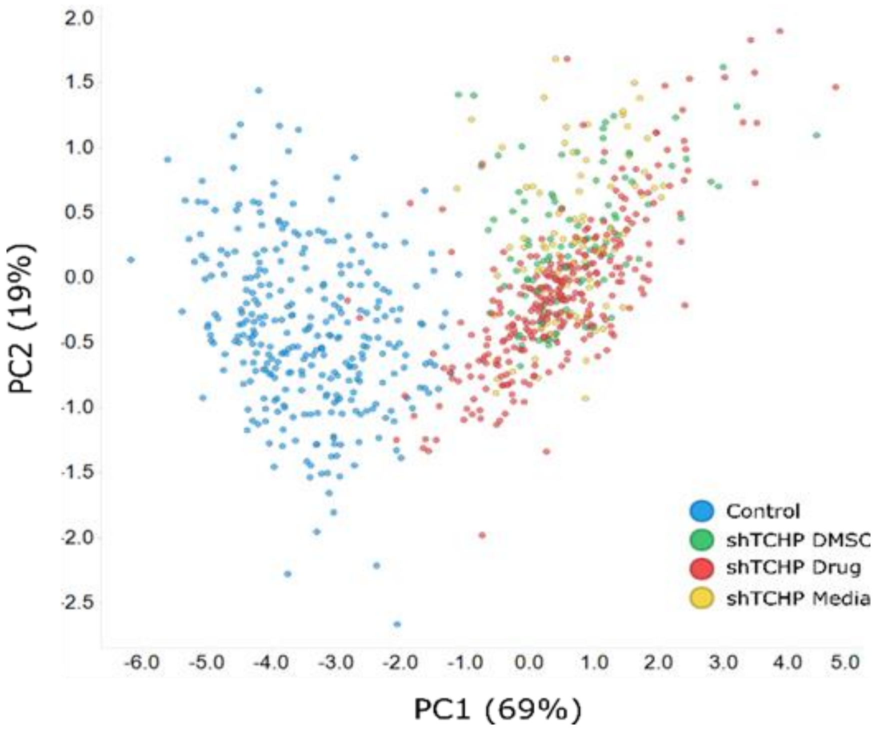

B

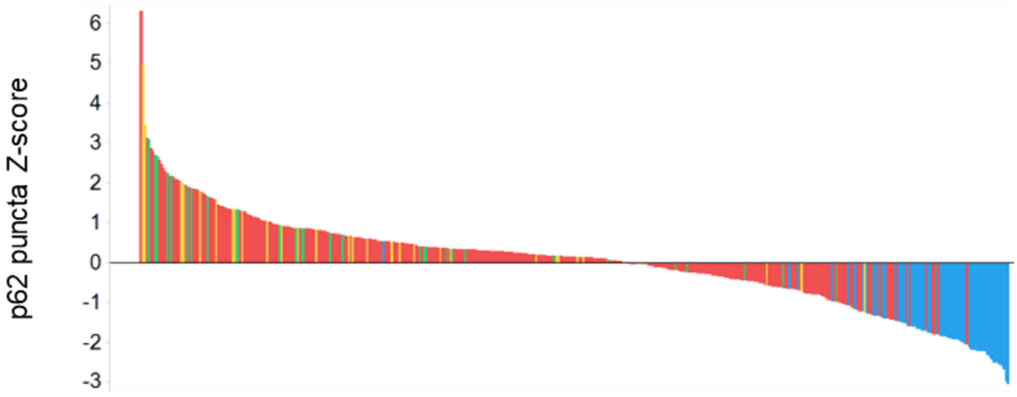

Appendix Figure S3: Phenotypic screening assay analysis

A, Principal component analysis of compound screen. Principal components (PC) 1 and 2 are shown and their respective variance indicated. **B**, Z-score ranking of cytoplasmic puncta of p62 immunostaining.

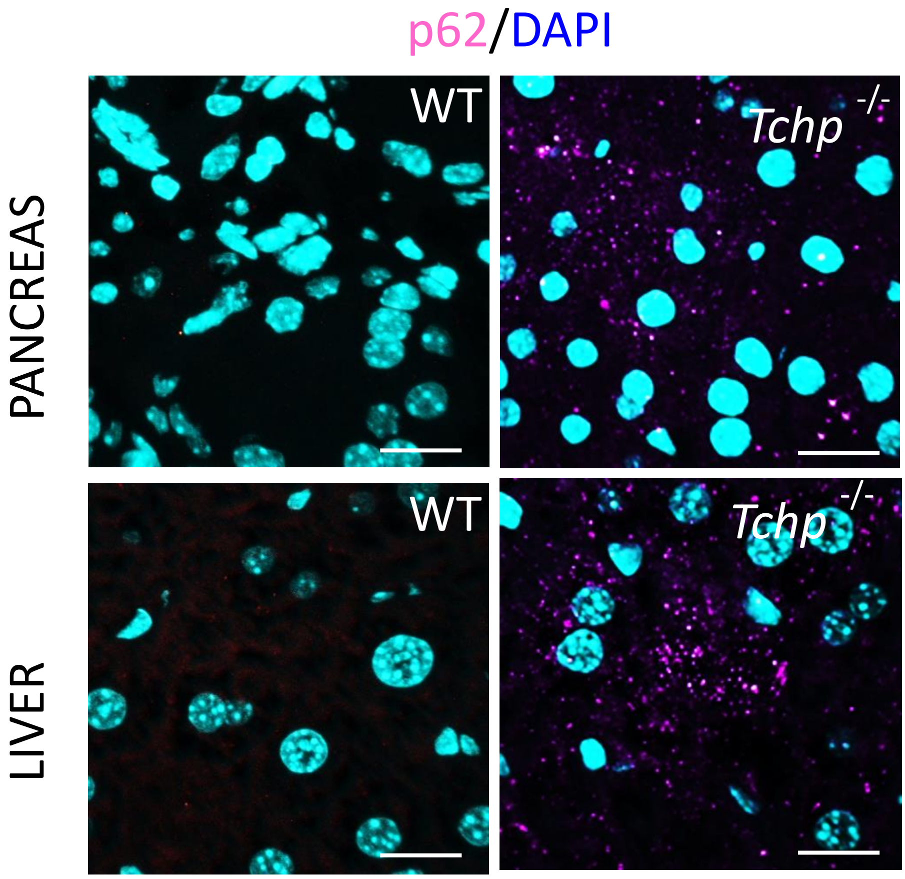

Appendix Figure S4: Characterization of Tchp knock-out mice
Representative images of the liver and pancreas of wild-type and Tchp knock-out mice stained for DAPI (blue) and p62 (red). Scale bars, 50µm.
