# Supplementary material for: Trichoplein binds PCM1 and controls endothelial cell function by regulating autophagy
Source: EMBO Rep. 2020 Apr 26;21(7):e48192. doi: 10.15252/embr.201948192 (PMC7332983; doi:10.15252/embr.201948192)
Supplement: Supplementary file 2 — Expanded View Figures PDF [file EMBR-21-e48192-s002.pdf]

Expanded View Figures

Figure EV1. TCHP knock-down affects endothelial cells function.

A Western blot anti-TCHP following knock-down of TCHP.
B Endothelial network formation on Matrigel was analysed by quantification of the total length of tube-like structures and number of meshes (unpaired *t*-test, $^{**}P = 0.0018$ and $^{**}P = 0.0014$ vs. control, respectively); *right panels*, representative microphotographs from Matrigel assay; scale bars, 100 μm.
C *Left panels*: representative images are showing the new microvessels positive for CD31 (green), in the implanted plugs. Scale bars, 50 and 25 μm for the inset. *Right panel*: quantification of the area of CD31 coverage in the Matrigel plugs mixed with TCHP siRNA or control oligos at 21 days after implantation; ($n = 5$ mice per group, unpaired *t*-test, $^{**}P = 0.0004$ vs. control).
D Effect of TCHP knock-down on HUVEC migration speed measured by electric cell-substrate impedance sensing (ECIS) (unpaired *t*-test, $^{**}P = 0.0013$ vs. control).
E Relative mRNA levels of TCHP and subset of genes. Graphs represent transcripts measured at 3 and 7 days post-TCHP knock-down (unpaired *t*-test vs. control; IL-1β: $^{**}P = 0.0048$; IL-6: $^{*}P = 0.015$; IL-8: $^{*}P = 0.0365$—3 days; $^{*}P = 0.0168$—7 days; MCP1: $^{*}P = 0.0223$; p16: $^{*}P = 0.0133$; p14: $^{**}P = 0.0073$; TCHP: $^{*}P = 0.0185$—3 days; $^{*}P = 0.0018$—7 days).
F Western Blot anti-p16.
G β-Galactosidase activity as reveal by the chromogenic β-Gal substrate X-Gal. Scale bars 25 μm. Lower panel: quantification (unpaired *t*-test, $^{**}P < 0.0001$ vs. control).
H Aggresome staining and quantification of protein aggregates; scale bars, 25 μm. Lower panel: quantification (unpaired *t*-test, $^{**}P < 0.0001$ vs. control).

Data information: Statistical analyses were performed on at least three independent experiments. Data are represented as mean ± SD.

© 2020 The Authors

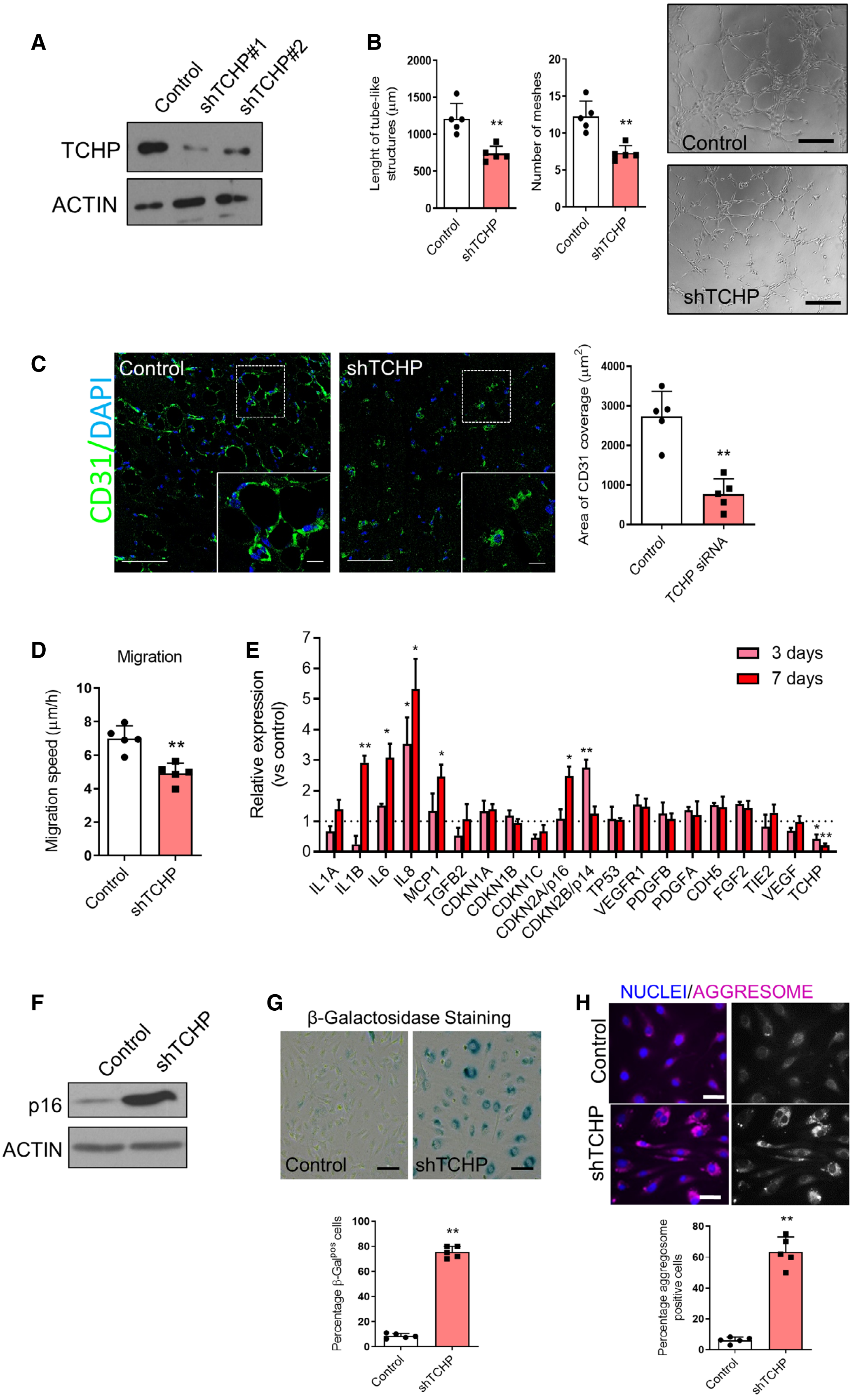

Figure EV1.

 © 2020 The Authors

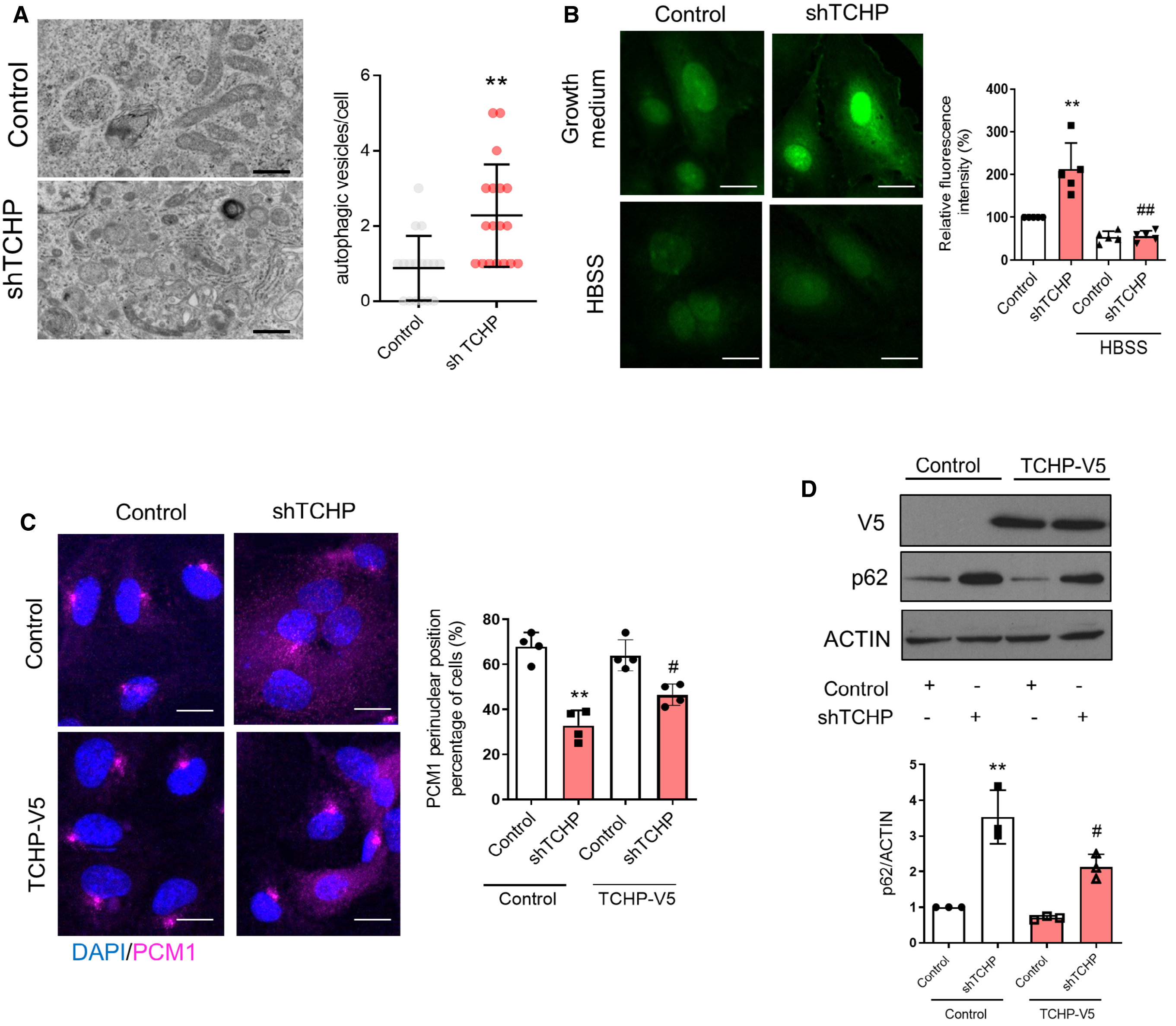

Figure EV2. Autophagic features in TCHP-depleted endothelial cells.

A Representative pictures from transmission electron microscopy analysis. Scale bars, 500 nm. Right panel: quantification of autophagic vacuoles (n = 16 cells, unpaired t-test; $^{**}P$ = 0.0010 vs. control).

B Representative pictures and quantification of HUVECs after Alexa 488 Click-iT® L-azidohomoalanine (AHA) labelling. Right panel: quantification (n = 50 cells, one-way ANOVA; $^{**}P$ = 0.0037 vs. control, $^{\#\#}P$ = 0.0005 vs. shTCHP). Scale bars, 25 μm.

C TCHP knock-down or control HUVECs were transduced with TCHP-V5 or control vectors and were stained for anti-PCM1 antibody. Panel below: quantification (n = 50 cells; unpaired t-test; $^{**}P$ = 0.0003 vs. control; $^{\#}P$ = 0.0166 vs. shTCHP). Scale bars, 25 μm.

D Western blot for anti-V5 and anti-p62 antibody (one-way ANOVA; $^{**}P$ = 0.0044; $^{\#}P$ = 0.0439 vs. TCHP-V5).

Data information: Statistical analyses were performed on at least three independent experiments. Data are mean ± SD.

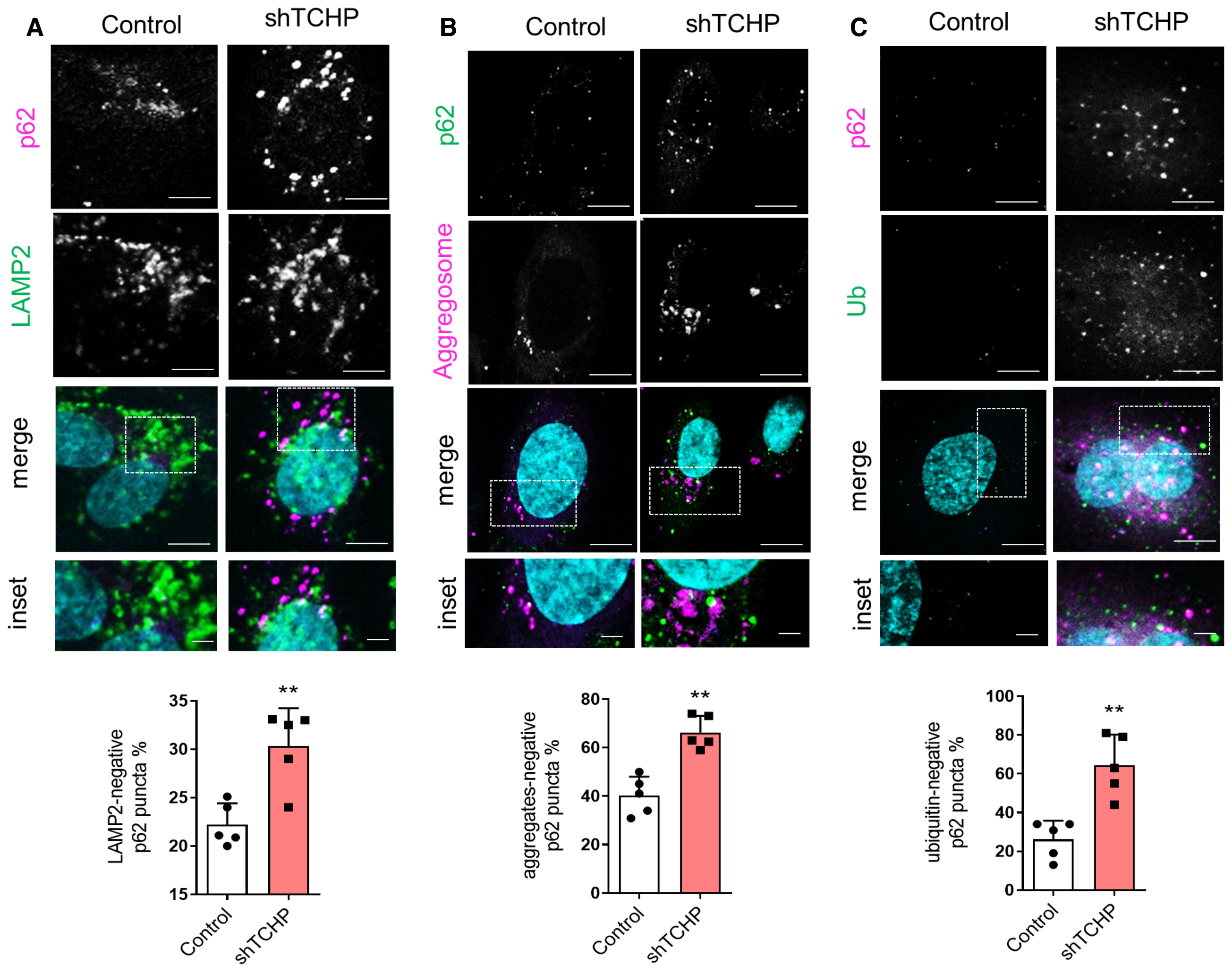

Figure EV3. Analysis of p62 localization in TCHP knock-down cells.

A–C Co-localization between (A), LAMP2 and p62, (B), aggregates and p62 and (C), ubiquitinated proteins and p62 in TCHP knock-down and control cells. Scale bars, 25 and 2 μm in the inset. Lower panels: quantification of the above panels (n = 80 cells, unpaired-test; A: **P = 0.0038 vs. control; B: **P = 0.0005 vs. control; C: **P = 0.0017 vs. control).

Data information: Statistical analyses were performed on at least three independent experiments. Data are represented as mean ± SD.

Figure EV4. Regulation of lysosomal distribution and activity in TCHP knock-down cells.

A Immunofluorescence for recycling (RAB11), early (EEA1), late (RAB7) endosomes and lysosome (LAMP1/2); scale bars, 25 μm.

B Quantitative analysis of LysoTracker Red by flow cytometry (unpaired t-test; **P = 0.0007 vs. control).

C Representative Western blot showing epidermal growth factor receptor (EGFR) levels during a pulse-chase experiment with EGF (20 nM) for the indicated times. *Right panel:* quantification, (n = 3, one-way ANOVA; EGF 60 min. **P = 0.0007 EGF 90 and 120 min. **P < 0.0001 vs. control time 0).

D Representative images showed TCHP knock-down and control cells stained for LAMP2 and αTubulin. Cells were analysed in growing medium (FM), after 2-h starvation (Starv.) or after 2-h starvation and replenishment of growing medium for 2 h (FM/Starv.) Scale bars, 10 μm. MT network was quantified as α-Tubulin intensity. LAMP2 radial integrated fluorescence intensity was used to quantify the distribution of lysosomes. The graph shows the percentage of cells showing cytoplasmic spread or perinuclear lysosome distribution (n = 20 cells, one-way ANOVA; nuclear: **P = 0.0031 vs. control FM, **P = 0.0026 vs. control FM/Starv. Spread: *P = 0.0195 vs. control FM, **P = 0.0001 vs. control FM/Starv) and MT intensity (n = 20 cells, one-way ANOVA; **P = 0.0056 vs. control FM).

E Western blot for anti-p62 in TCHP knock-down and control cells after serum starvation.

Data information: Statistical analyses were performed on at least three independent experiments. Data are represented as mean ± SD.

 © 2020 The Authors

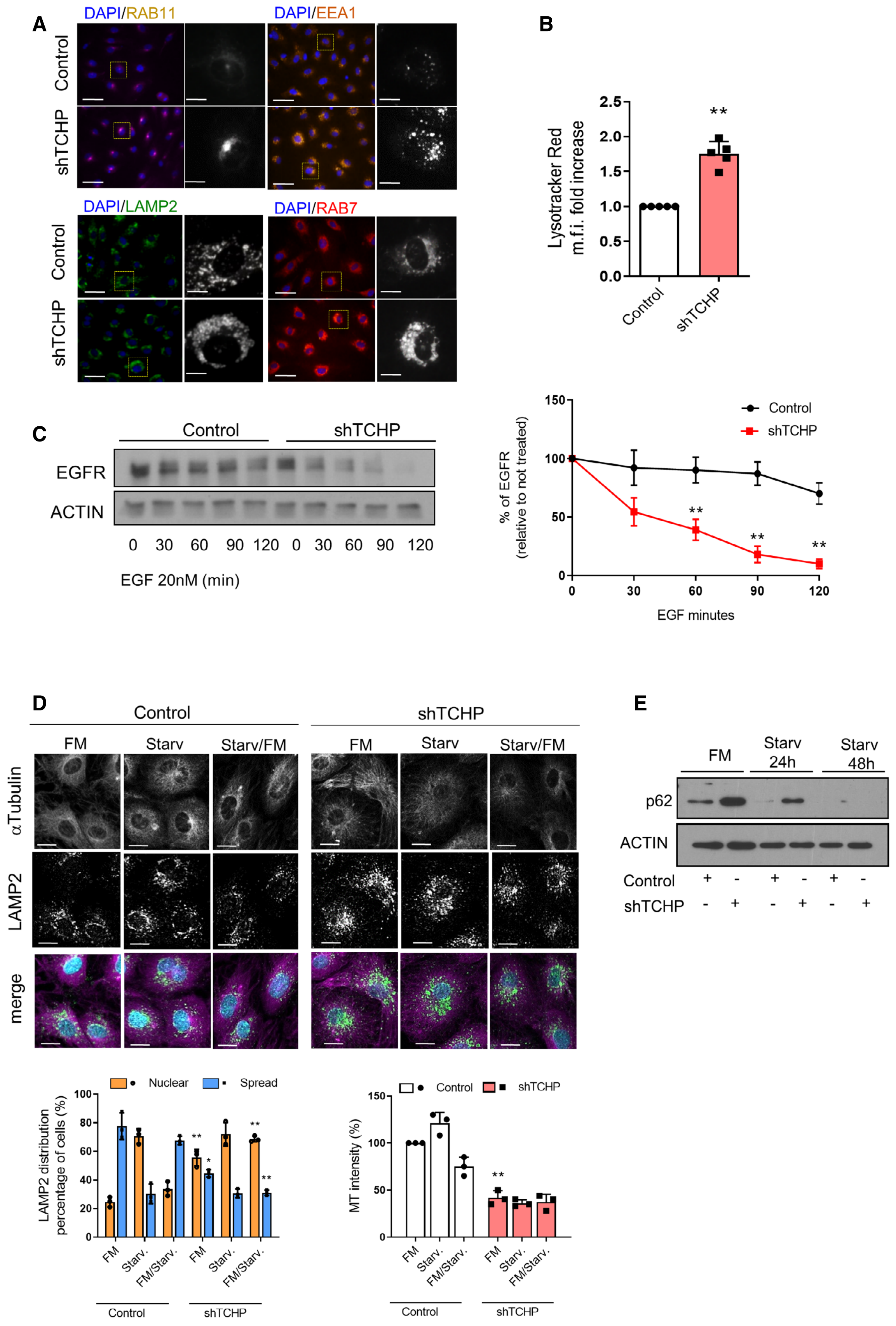
A
DAPI/RAB11
DAPI/EEA1
Control
shTCHP
DAPI/LAMP2
DAPI/RAB7
B
Lysotracker Red m.f.i. fold increase
2.5
2.0
1.5
1.0
0.5
0.0
**
Control
shTCHP
C
Control
shTCHP
EGFR
ACTIN
0 30 60 90 120 0 30 60 90 120
EGF 20nM (min)
% of EGFR (relative to not treated)
150
100
50
0
30
60
90
120
EGF minutes
D
FM
Starv
Starv/FM
αTubulin
LAMP2
merge
Nuclear
Spread
LAMP2 distribution percentage of cells (%)
Starv.
FM/Starv.
MT intensity (%)
E
Starv 24h
Starv 48h
p62
+ - + - + -
- + - + - +

Figure EV4.

Figure EV5. Identification of the mechanism of p62 accumulation.

A Representative images for p62 of control and TCHP knock-down HUVECs treated with BAY11-7082 (300 nM) or TYRPHOSTIN AG1288 (300 nM) or SB202190 (300 nM) or vehicle (DMSO) for 48 h. Scale bars, 50 μm.

B, C (B) Expression of IL-6, IL-8 and IL-1β (one-way ANOVA; IL-8: $^{**}P$ = 0.008 vs. control DMSO; $^{\#}P$ = 0.0198 (BAY) $^{\#}P$ = 0.01 (AG) vs. shTCHP DMSO; IL-6: $^{**}P$ = 0.0023 vs. control DMSO; $^{\#}P$ = 0.0063 (BAY) $^{\#}P$ = 0.0108 (AG) vs. shTCHP DMSO; IL-1β: $^{**}P$ = 0.0011 vs. control DMSO; $^{\#}P$ = 0.0004 (BAY) $^{\#}P$ = 0.0015 (AG) vs. shTCHP DMSO) and (C), migration speed was measured (one-way ANOVA; $^{**}P$ = 0.0003 vs. control DMSO; $^{\#}P$ = 0.0451 vs. shTCHP DMSO).

D Western blot analysis for anti-phosphor-NF-κB (S536), total NF-κB, in TCHP knock-down and control cells treated with BAY or vehicle. *Below panel:* quantification (one-way ANOVA; $^{**}P$ = 0.0014 vs. control DMSO; $^{\#}P$ = 0.0293 vs. shTCHP DMSO).

E p62 expression in TCHP knock-down and control cells treated with BAY or vehicle (one-way ANOVA; $^{**}P$ = 0.0004 vs. control DMSO; $^{\#}P$ = 0.0243 vs. shTCHP DMSO).

F p62 expression in TCHP knock-down and control cells treated with siRELA or control (one-way ANOVA; $^{**}P$ = 0.0023 vs. control siRNA; $^{\#}P$ = 0.0127 vs. shTCHP siRELA).

G ChIP-qPCR analysis confirms the of NF-κB p65 enrichment to IκBα and p62 promoter in TCHP knock-down cells (unpaired *t*-test; $^{**}P$ = 0.0057 and $^{**}P$ = 0.0037 vs. control, respectively).

Data information: Statistical analyses were performed on at least three independent experiments. Data are represented as mean ± SD.

 © 2020 The Authors

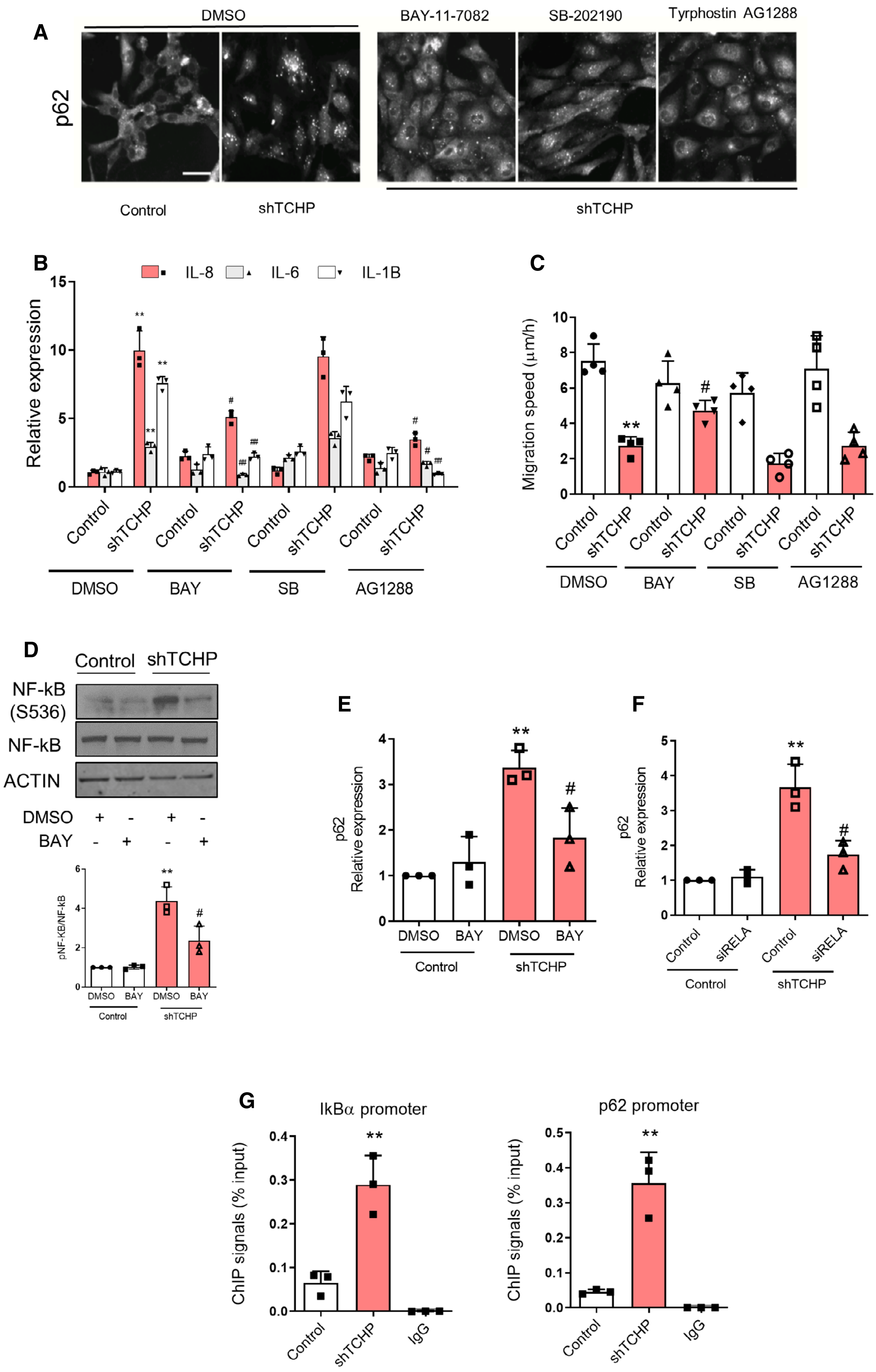
A
DMSO
BAY-11-7082
SB-202190
Tyrphostin AG1288
p62
Control
shTCHP
shTCHP
B
IL-8
IL-6
IL-1B
Relative expression
Control
shTCHP
DMSO
BAY
SB
AG1288
C
Migration speed (μm/h)
Control
shTCHP
DMSO
BAY
SB
AG1288
D
Control
shTCHP
NF-kB (S536)
NF-kB
ACTIN
DMSO
BAY
pNF-KB/NF-kB
E
p62 Relative expression
DMSO
BAY
Control
shTCHP
F
p62 Relative expression
Control
siRELA
Control
shTCHP
G
IkBα promoter
ChIP signals (% input)
Control
shTCHP
IgG
p62 promoter
ChIP signals (% input)
Control
shTCHP
IgG

Figure EV5.
