# Supplementary material for: Trichoplein binds PCM1 and controls endothelial cell function by regulating autophagy
Source: EMBO Rep. 2020 Apr 26;21(7):e48192. doi: 10.15252/embr.201948192 (PMC7332983; doi:10.15252/embr.201948192)
Supplement: Supplementary file 3 — Table EV1 [file EMBR-21-e48192-s003.docx]

**Table EV1:** Mass spectrometry analysis of proteins co-immunoprecipitated with TCHP-FLAG in HEK293 cell extracts.

| Accession | Peptide count | Unique peptides | Confidence score | Anova (p-) | Max fold change | Gene Symbol | Description |
| --- | --- | --- | --- | --- | --- | --- | --- |
| P16278 | 6 | 6 | 391.84 | 0.0074 | 13.8920 | GLB1 | Beta-galactosidase |
| B5ME19 | 2 | 2 | 59.37 | 0.0172 | 13.3794 | EIF3CL | Eukaryotic translation initiation factor 3 subunit C-like protein |
| E7EQ29 | 2 | 2 | 49.36 | 0.0477 | 13.2170 | GLB1 | Beta-galactosidase |
| H0Y4R1 | 5 | 5 | 239.44 | 0.0106 | 10.3089 | IMPDH2 | Inosine-5'-monophosphate dehydrogenase 2 (Fragment) |
| Q93008 | 9 | 9 | 322.58 | 0.0301 | 10.1639 | USP9X | Probable ubiquitin carboxyl-terminal hydrolase |
| Q5JZH0 | 3 | 3 | 190.15 | 0.0254 | 9.7011 | CTSA | Lysosomal protective protein (Fragment) |
| A6NNN6 | 5 | 5 | 225.79 | 0.0103 | 8.3449 | PCM1 | Pericentriolar material 1 protein |
| Q86YT6 | 6 | 6 | 237.02 | 0.0310 | 8.0604 | MIB1 | E3 ubiquitin-protein ligase MIB1 |
| Q9BT92 | 16 | 15 | 1077.52 | 0.0127 | 8.0441 | TCHP | Trichoplein keratin filament-binding protein |
| F5GY55 | 5 | 4 | 199.65 | 0.0239 | 7.4310 | DDB1 | DNA damage-binding protein 1 |
| E7ETA6 | 8 | 8 | 533.59 | 0.0102 | 7.3776 | PCM1 | Pericentriolar material 1 protein |
| A0A087X253 | 2 | 2 | 77.36 | 0.0297 | 5.9613 | AP2B1 | AP-2 complex subunit beta |
| O75592 | 2 | 2 | 64.27 | 0.0209 | 5.2015 | MYCBP2 | E3 ubiquitin-protein ligase MYCBP2 |
| Q8N163 | 3 | 3 | 95.01 | 0.0343 | 4.8284 | CCAR2 | Cell cycle and apoptosis regulator protein 2 |
| P07900 | 5 | 2 | 212.31 | 0.0166 | 3.3960 | HSP90AA | Heat shock protein HSP 90-alpha |
| P11388 | 11 | 7 | 422.86 | 0.0407 | 3.1956 | TOP2A | DNA topoisomerase 2-alpha |
| E7EMC7 | 3 | 3 | 146.59 | 0.0419 | 3.1848 | SQSTM1 | Sequestosome-1 |
| E7ERL0 | 3 | 3 | 113.7 | 0.0124 | 2.8382 | NME1 | Nucleoside diphosphate kinase A |
| P16989 | 5 | 3 | 270.7 | 0.0086 | 2.8340 | YBX3 | Y-box-binding protein 3 |
| Q86UE4 | 4 | 4 | 273.45 | 0.0309 | 2.6732 | MTDH | Protein LYRIC |
| Q96AG4 | 8 | 8 | 493.3 | 0.0147 | 2.5912 | LRRC59 | Leucine-rich repeat-containing protein 59 |
| P56192 | 2 | 2 | 92.19 | 0.0430 | 2.5739 | MARS | Methionine--tRNA ligase, cytoplasmic |
| H7BY10 | 5 | 5 | 198.63 | 0.0494 | 2.5139 | RPL23A | 60S ribosomal protein L23a (Fragment) |
| P07814 | 5 | 5 | 223.42 | 0.0471 | 2.4357 | EPRS | Bifunctional glutamate/proline--tRNA ligase |
| P62851 | 13 | 13 | 880.15 | 0.0421 | 2.3574 | RPS25 | 40S ribosomal protein S25 |
| P05198 | 4 | 4 | 148.59 | 0.0337 | 2.2545 | EIF2S1 | Eukaryotic translation initiation factor 2 subunit 1 |
| A0A0A0MSX9 | 4 | 4 | 177.23 | 0.0363 | 2.2359 | IARS | Isoleucine--tRNA ligase, cytoplasmic |
| H7C1M2 | 3 | 3 | 113.31 | 0.0309 | 2.1911 | SON | Protein SON (Fragment) |
| C9JNW5 | 6 | 6 | 313.54 | 0.0020 | 2.1519 | RPL24 | 60S ribosomal protein L24 |
| Q5JNZ5 | 2 | 2 | 88.01 | 0.0098 | 2.0239 | RPS26P11 | Putative 40S ribosomal protein S26-like 1 |
| A0A087WUT6 | 15 | 15 | 895.99 | 0.0010 | 1.9783 | EIF5B | Eukaryotic translation initiation factor 5B |
| P62753 | 7 | 7 | 452.58 | 0.0406 | 1.9533 | RPS6 | 40S ribosomal protein S6 |
| B5MCW2 | 2 | 2 | 68.64 | 0.0435 | 1.9386 | RPL3 | 60S ribosomal protein L3 (Fragment) |
| P78527 | 22 | 22 | 927.74 | 0.0122 | 1.9132 | PRKDC | DNA-dependent protein kinase catalytic subunit |
| A0A087WXM6 | 5 | 5 | 179.48 | 0.0308 | 1.8681 | RPL17 | 60S ribosomal protein L17 (Fragment) |
| A0A024QZP7 | 4 | 3 | 151.96 | 0.0476 | 1.8671 | CDK1 | Cyclin-dependent kinase 1 |
| A0A0G2JIC2 | 2 | 2 | 56.05 | 0.0217 | 1.8155 | ABCF1 | ATP-binding cassette sub-family F member 1 |
| Q7KZF4 | 2 | 2 | 79.14 | 0.0026 | 1.7306 | SND1 | Staphylococcal nuclease domain-containing protein 1 |
| O00203 | 14 | 14 | 864.16 | 0.0062 | 1.6903 | AP3B1 | AP-3 complex subunit beta-1 |
| P09874 | 35 | 35 | 2558.68 | 0.0455 | 1.6615 | PARP1 | Poly [ADP-ribose] polymerase 1 |
| P07437 | 25 | 5 | 1943.11 | 0.0392 | 1.6381 | TUBB | Tubulin beta chain |
| P14868 | 2 | 2 | 66.38 | 0.0063 | 1.6269 | DARS | Aspartate--tRNA ligase |
| P12814 | 8 | 2 | 274.02 | 0.0105 | 1.5408 | ACTN1 | Alpha-actinin-1 |
| Q14683 | 4 | 3 | 120.19 | 0.0375 | 1.5385 | SMC1A | Structural maintenance of chromosomes protein 1A |
| P22626 | 4 | 4 | 232.72 | 0.0017 | 1.5234 | HNRNPA2B1 | Heterogeneous nuclear ribonucleoproteins A2/B1 |
| K7EMH1\| | 7 | 6 | 409.08 | 0.0332 | 1.5132 | RPL22 | 60S ribosomal protein L22 (Fragment) |
