# Supplementary material for: Trichoplein binds PCM1 and controls endothelial cell function by regulating autophagy
Source: EMBO Rep. 2020 Apr 26;21(7):e48192. doi: 10.15252/embr.201948192 (PMC7332983; doi:10.15252/embr.201948192)
Supplement: Supplementary file 4 — Table EV2 [file EMBR-21-e48192-s004.docx]

| Chemical Name | Target | [Drug] uM | Cell Count | Cytoplasmic Granule Count | Prediction Score |
| --- | --- | --- | --- | --- | --- |
| Splitomicin | SIRT-2 inhibitor | 3.3 | 211 | 0.53 | 0.84 |
| Apicidin | HDAC inhibitor | 3.3 | 170 | 0.42 | 0.79 |
| BAY 11-7082 | IKK pathway | 0.3 | 237 | 0.36 | 0.78 |
| SB-202190 | p38 MAPK | 0.3 | 267 | 0.39 | 0.72 |
| Terreic acid | BTK | 3.3 | 174 | 0.50 | 0.70 |
| TYRPHOSTIN AG 1288 | Tyrosine kinases | 3.3 | 215 | 0.31 | 0.69 |
| Aminoresveratrol sulfate | SIRT1 activator | 0.3 | 206 | 0.42 | 0.68 |
| Daidzein | Negative control for Genistein | 0.3 | 199 | 0.46 | 0.68 |
| Epigallocatechin gallate | MMPs | 3.3 | 220 | 0.50 | 0.66 |
| N-Ethylmaleimide | Cysteine proteases | 3.3 | 233 | 0.59 | 0.61 |
| LY 294002 | PI 3-K | 3.3 | 118 | 0.46 | 0.57 |
| E-64-C | Calpain; Cathepsins; Papain | 0.3 | 246 | 0.53 | 0.57 |
| Calpain Inhibitor II (ALLM) | Calpain; Cathepsins L, B | 0.3 | 254 | 0.63 | 0.56 |
| Tranylcypromine hemisulfate | Lysine demethylase inhibitor | 3.3 | 208 | 0.60 | 0.53 |
| Daidzein | Negative control for Genistein | 3.3 | 196 | 0.65 | 0.53 |
| GW 5074 | cRAF | 3.3 | 212 | 0.65 | 0.53 |
| TYRPHOSTIN 46 | EGFRK, PDGFRK | 3.3 | 203 | 0.54 | 0.52 |
| Lavendustin A | EGFRK | 3.3 | 215 | 0.71 | 0.52 |
| Nullscript | Scriptaid Neg control | 3.3 | 177 | 0.72 | 0.50 |
| Suramin·6Na | SIRT1 inhibitor | 3.3 | 218 | 0.64 | 0.47 |
| Roscovitine | CDK | 0.3 | 219 | 0.65 | 0.46 |
| Z-PLG-NHOH | MMPs | 0.3 | 250 | 0.60 | 0.45 |
| Piceatannol | Syk | 0.3 | 273 | 0.76 | 0.44 |
| ML-9Â·HCl | MLCK | 0.3 | 241 | 0.68 | 0.43 |
| SB-203580 | p38 MAPK | 3.3 | 269 | 0.81 | 0.42 |

**Table EV2:** Analysis of compounds reversing p62 accumulation in TCHP knock-down cells
