# Supplementary figures and images for: Trichoplein binds PCM1 and controls endothelial cell function by regulating autophagy
Source: EMBO Rep. 2020 Apr 26;21(7):e48192. doi: 10.15252/embr.201948192 (PMC7332983; doi:10.15252/embr.201948192)

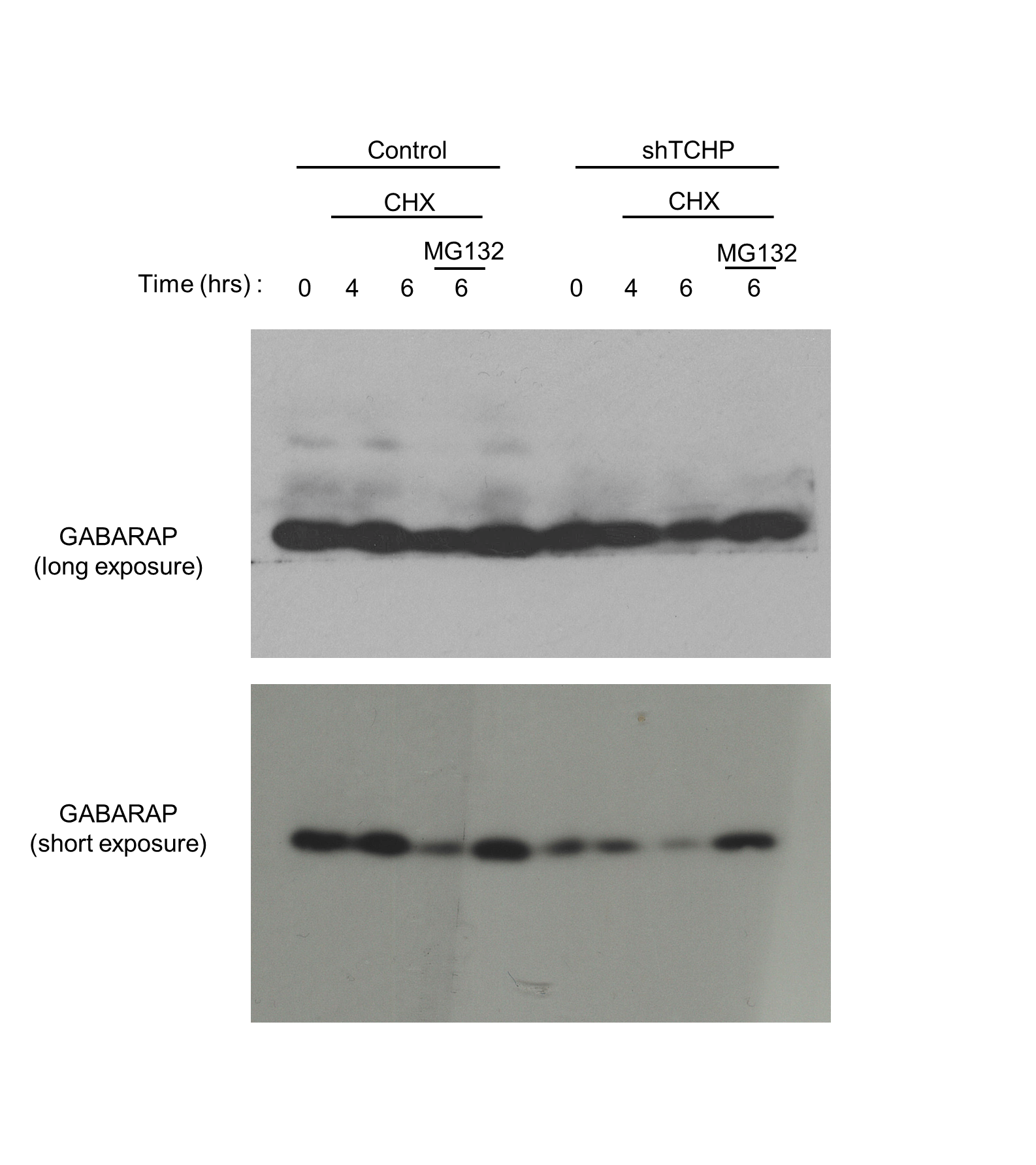

Supplement: Supplementary file 6 — Source Data for Figure 2 [file EMBR-21-e48192-s005.tif]
